# Cutaneous Langerhans cell histiocytosis and other systemic inflammatory or autoimmune disease manifestations in the setting of clonal hematopoiesis

**DOI:** 10.1002/jha2.974

**Published:** 2024-07-04

**Authors:** Giby V. George, Jane Liesveld, Siba El Hussein, Audrey N. Jajosky

**Affiliations:** ^1^ Department of Pathology and Laboratory Medicine University of Rochester Medical Center Rochester New York USA; ^2^ Wilmot Cancer Institute University of Rochester Medical Center Rochester New York USA; ^3^ Department of Pathology The University of Vermont Larner College of Medicine Burlington Vermont USA

**Keywords:** CH, CHIP, clonal hematopoiesis, LCH, SIAD

## Abstract

The clinical manifestations and pathophysiology of clonal hematopoiesis (CH)‐associated immunological dysfunction are poorly understood. We describe an elderly woman with CH who developed various systemic inflammatory or autoimmune diseases (SIADs), including cutaneous Langerhans cell histiocytosis (LCH) and temporal arteritis. Sequencing of the LCH revealed somatic oncogenic mutations in *MAP2K1*, *IDH2*, and *SRSF2*, with enrichment of the latter two in her peripheral blood at high allele frequencies. These findings raise concern for the future development of a myeloid malignancy. Given the mounting evidence for adult‐onset autoinflammatory conditions caused by somatic blood mutations, we suspect CH‐mediated immune dysregulation is contributing to her multi‐organ involvement by a combination of SIADs.

## 1 INTRODUCTION

Systemic inflammatory or autoimmune diseases (SIADs) are well‐described in patients with myeloid malignancies. More recently, clinical and laboratory evidence of immune system dysregulation is increasingly recognized in patients with clonal hematopoiesis (CH) who do not have hematologic malignancies. The full clinical spectrum and underlying pathophysiology of CH‐associated immunological dysfunction are still being elucidated. Here we describe the clinicopathologic and molecular findings in an elderly woman with a wide variety of presumed CH‐associated SIADs.

## 2 CASE PRESENTATION

**FIGURE 1:**
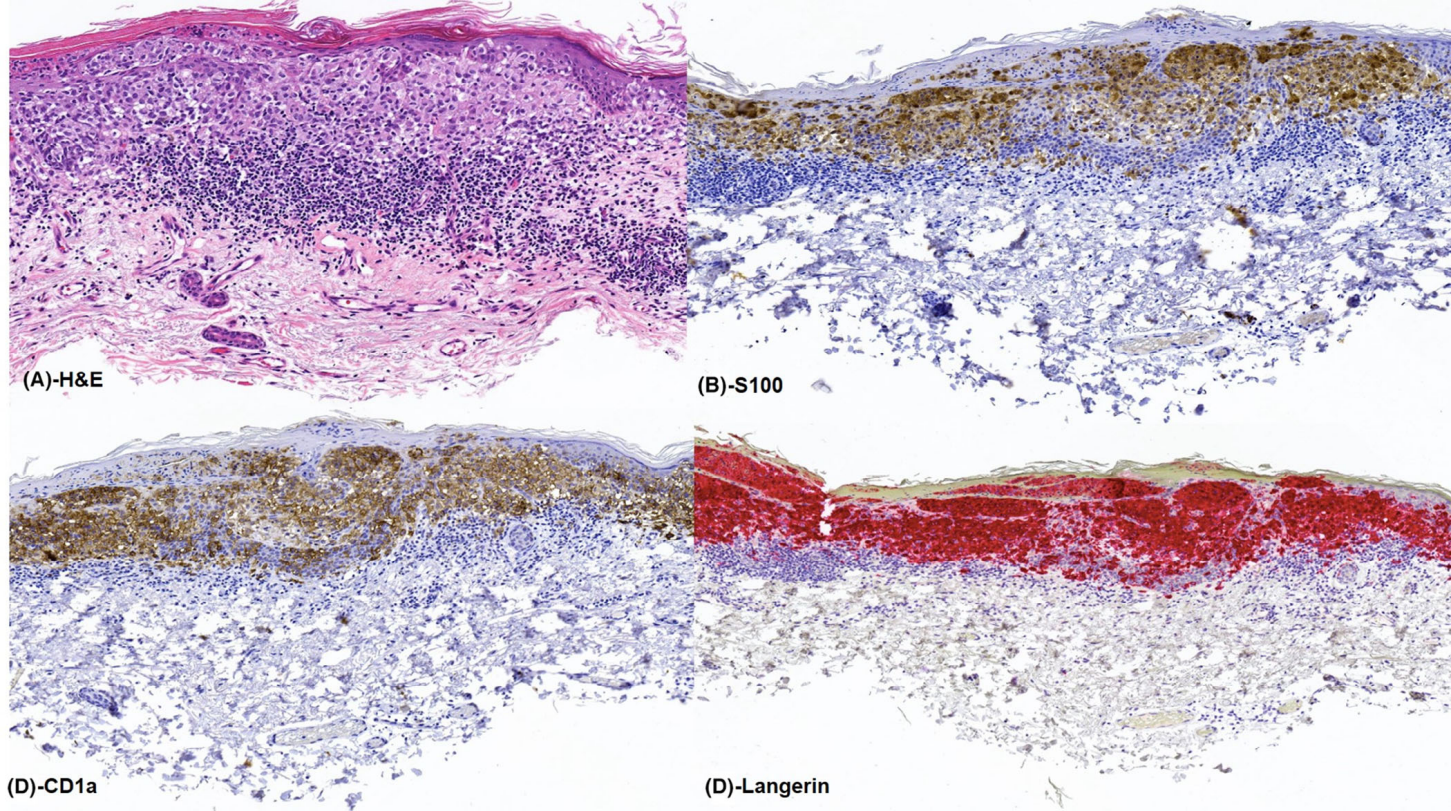
(A) Microscopic examination of the skin showing a proliferation of Langerhans cells in the epidermis and dermis, surrounded by a dense lymphocytic infiltrate. (B) The Langerhans cells are positive for S100, (C) CD1a, and (D) langerin, supporting a diagnosis of Langerhans cell histiocytosis (LCH).

**FIGURE 2:**
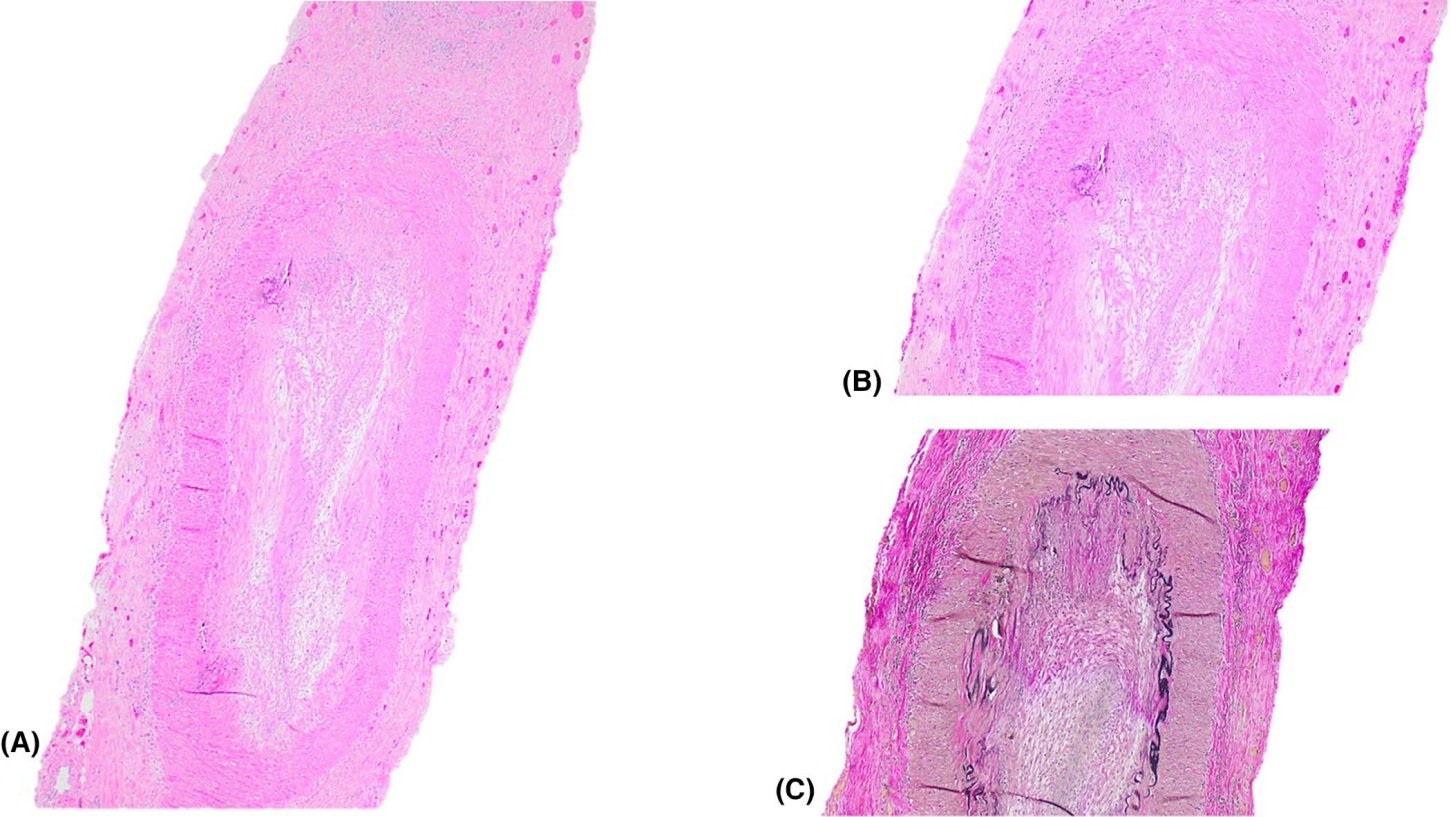
(A) Microscopic examination of the temporal artery showing luminal occlusion, thickening of the intima, and transmural inflammation with a conspicuous giant cell. (B) Higher power magnification of the transmural inflammation and giant cell. (C) Elastin stain highlighting the discontinuity of the internal elastic lamina.

An 89‐year‐old woman with a 15‐pack‐year smoking history, hereditary protein S deficiency, chronic kidney disease, and a prior history of SIADs – including rheumatoid arthritis in remission, chronic inflammatory demyelinating polyneuropathy, and vulvar malakoplakia – presented with a long‐standing skin rash on her trunk. A biopsy revealed cutaneous Langerhans cell histiocytosis (LCH) (Figure 1). Shortly thereafter, she developed sudden painless right vision loss leading to unilateral blindness that was preceded by a 1‐month history of bilateral jaw pain with chewing. A biopsy was consistent with temporal arteritis (Figure 2).

Targeted DNA‐based next‐generation sequencing (NGS) of her cutaneous LCH using ThermoFisher's 35‐gene Oncomine Focus Assay and 45‐gene Oncomine Myeloid Assay GX revealed oncogenic mutations in *MAP2K1* p.C121S (variant allele frequency [VAF] = 4%), *IDH2* p.R140Q (VAF = 8%), and *SRSF2* p.P95L (VAF = 6%). These VAFs are consistent with the reported 20% neoplastic cellularity. Since *IDH2* mutations have not been reported in LCH but are well‐described in hematologic malignancies, targeted DNA‐based NGS was also performed on her peripheral blood using the 34‐gene Illumina TruSight Myeloid Panel. Both the *IDH2* p.R140Q (VAF = 46%) and *SRSF2* p.P95L (VAF = 51%) mutations were highly enriched in her blood, consistent with CH. The *MAP2K1* variant was not detected in the blood, suggesting it is a specific LCH driver mutation. Of note, sequencing of the temporal artery biopsy was not performed.

Fortunately, after treatment with high‐dose steroids for the temporal arteritis, she suffered no further vision loss. Her cutaneous LCH also responded to steroid and methotrexate therapy. She was later placed on the anti‐IL6R biologic ‘tocilizumab’ as a steroid‐sparing agent. She has not yet had a bone marrow biopsy given her normal peripheral blood counts (hemoglobin: 12.2 g/dL, platelet count: 235 THOU/µL, absolute lymphocyte count: 0.6 THOU/µL, absolute neutrophil count: 3.7 THOU/µL, absolute monocyte count: 0.6 THOU/µL, absolute eosinophil count: 0 THOU/µL, and absolute basophil count: 0 THOU/µL). However, she is being closely monitored by her hematologist‐oncologist.

## 3 DISCUSSION

The discovery of CH in this elderly woman with a wide spectrum of SIADs supports an intriguing link between somatic blood mutations and her adult‐onset rheumatologic disorders. Mechanistically, disrupted *SRSF2*‐mediated splicing may be driving aberrant antigen presentation by bone marrow‐derived dendritic cells in her skin and other organs. Causal relationships between somatic blood mutations and SIADs have been established in patients with hematologic malignancies and conditions like VEXAS (vacuoles, E1 enzyme, X‐linked, auto‐inflammatory, somatic) syndrome [1].

Although this patient has not yet had a bone marrow biopsy, the high‐level somatic *IDH2* and *SRSF2* variants present in nearly all of her circulating white blood cells raise concern for the future development of a myeloid malignancy, such as a myelodysplastic neoplasm (previously myelodysplastic syndrome [MDS]) or acute myeloid leukemia [AML]). The genomic landscapes of MDS and myelodyplastic/myeloproliferative (MDS/MPN) overlap syndromes such as chronic myelomonocytic leukemia (CMML) overlap with that of CH and include mutations in genes affecting epigenetic regulation (*TET2* and *DNMT3A*), histone modification (*ASXL1* and *EZH2*), splicing factors (*SRSF2*, *SF3B1*, *U2AF1*, and *ZRSR2*), signal transduction (*NRAS*, *KRAS*, *CBL*, *PTPN11*, and *JAK2*), and nucleosome assembly (*SETBP1* and *RUNX1*) [2]. Although the rate of progression of CH to frank hematologic malignancy is only 0.5%–1% per year, the potential for progression increases with the presence of a sizable clone or multiple genetic aberrations [2, 3]. Specifically, mutations in the following genes have been associated with an increased risk of disease progression: *TP53*, *U2AF1*, *SRSF2*, *IDH2*, *IDH1*, *SF3B1*, and *ASXL1* [2]. Remarkably, evaluation of the premalignant molecular landscape of 212 patients who later developed AML in the Women's Health Initiative cohort found that all subjects with mutations in *TP53* and *IDH1*, and *IDH2* eventually developed AML during follow‐up (median time: 9.6 years) [4]. Increased surveillance of patients harboring these CH may therefore be warranted.

Although immunological abnormalities in MDS patients have been recognized for decades, only recently have the mechanistic underpinnings of MDS‐associated SIADs begun to be understood [3, 5]. Up to 25% of MDS/CMML cases co‐occur with SIADs [5, 6]. Autoimmune disorders are more frequent in patients with MDS and, reciprocally, patients with autoimmune disorders have an increased risk of developing MDS, although it is unclear to what extent medications contribute to the pathogenesis [5]. Aberrancies in both the innate and adaptive immune systems leading to lymphocyte and myeloid‐cell dysregulation play a role in MDS and CMML along with cytokine abnormalities [3, 5, 7].

To investigate the mutational and immunological landscape of MDS/CMML patients with SIADs, Zhao et al. performed a retrospective study and found mutations affecting *TET2*/*IDH* (95% [50/85] vs. 38% [122/319], *p* < 0.01) and *SRSF2* (31% [26/85] vs. 15% [47/319], *p* < 0.01) to be more frequent in MDS/CMML patients with SIADs than those without SIADs [6]. To understand the prevalence of VEXAS syndrome among males in this population, they examined the frequency of somatic *UBA1* mutations and found only 12% (4/33) had concomitant MDS and *UBA1* mutations [8]. Whereas in VEXAS syndrome, *UBA1* mutations alone are thought to drive both the inflammatory and MDS phenotypes, in *UBA1*‐wildtype patients with MDS/CMML, early somatic mutations in *TET2/IDH1* (and/or *SRSF2*) are thought to contribute to the MDS/CMML and SIAD components [7, 8]. Importantly, no statistically significant difference has been observed in overall survival or progression‐free survival between MDS/CMML patients with SIADs and those without [6, 9]. Treatment for high‐risk MDS and CMML often includes supportive care, systemic steroids, hypomethylating agents such as azacytidine (AZA), and possible allogeneic hematopoietic stem cell transplantation [9, 10]. Notably, AZA has demonstrated sustained and steroid‐sparing response in the treatment of SIADs associated with MDS and CMML [11].

Patients with myeloid malignancies often present with a wide range of cutaneous manifestations, which may precede a diagnosis of a hematologic malignancy, occur at the time of diagnosis or arise later during the course of disease [12, 13]. Within this setting, dermatologic findings may be classified as specific, attributable to direct malignant infiltration of the skin, or non‐specific [13]. CMML is among the most common myeloid disorders associated with a wide range of cutaneous manifestations [12, 14]. To our knowledge, however, only a few reports of cutaneous LCH in the setting of myeloid malignancies have been described in the literature [15, 16, 17, 18]. Although data regarding cutaneous findings in patients with CH have not been described to date, recent publications provide further evidence that cutaneous manifestations of bone marrow pathologies share common clonal origins [18, 19]. Thus, comprehensive molecular analyses may be warranted in patients with cutaneous manifestations to identify associated myeloid neoplasms or emerging pre‐leukemic clones [18].

In our case, we were able to successfully demonstrate the clonal relationship between the patient's cutaneous LCH and peripheral blood findings. Although this patient does not meet the diagnostic criteria for a hematologic malignancy, she will be closely monitored given her concerning mutational profile and immune dysregulation that may someday manifest as a myeloid malignancy. Notably, this is the first reported case of LCH harboring an *IDH2* mutation, raising the potential therapeutic utility of a Food and Drug Administration‐approved IDH2 inhibitor in this entity. Additional molecular studies are necessary to further understand the prevalence, pathogenesis, and prognosis of CH associated with SIADs.

## AUTHOR CONTRIBUTIONS

Giby V. George wrote the manuscript with input from Audrey N. Jajosky. Jane Liesveld and Siba El Hussein reviewed the manuscript. All authors approved the final version of the manuscript.

## CONFLICT OF INTEREST STATEMENT

The authors declare no conflict of interest.

## FUNDING INFORMATION

The study was not supported by a sponsor or funding agency.

## ETHICS STATEMENT

The authors have confirmed that an ethical approval statement is not required for this submission.

## PATIENT CONSENT STATEMENT

The authors have confirmed that a patient consent statement is not needed for this submission.

## CLINICAL TRIAL REGISTRATION

The authors have confirmed that clinical trial registration is not required for this submission.

## Data Availability

Data sharing is not applicable to this article as no datasets were generated or analyzed during the current study.
